# Slope Estimation during Normal Walking Using a Shank-Mounted Inertial Sensor

**DOI:** 10.3390/s120911910

**Published:** 2012-08-29

**Authors:** Antonio M. López, Diego Álvarez, Rafael C. González, Juan C. Álvarez

**Affiliations:** Multisensor Systems and Robotics Laboratory, University of Oviedo, Campus de Viesques, ed. 2, 33204, Gijón, Asturias, Spain; E-Mails: dalvarez@uniovi.es (D.A.); rcgonzalez@uniovi.es (R.C.G.); juan@uniovi.es (J.C.A.)

**Keywords:** slope estimation, shank, inertial sensors, gait, tilt

## Abstract

In this paper we propose an approach for the estimation of the slope of the walking surface during normal walking using a body-worn sensor composed of a biaxial accelerometer and a uniaxial gyroscope attached to the shank. It builds upon a state of the art technique that was successfully used to estimate the walking velocity from walking stride data, but did not work when used to estimate the slope of the walking surface. As claimed by the authors, the reason was that it did not take into account the actual inclination of the shank of the stance leg at the beginning of the stride (mid stance). In this paper, inspired by the biomechanical characteristics of human walking, we propose to solve this issue by using the accelerometer as a tilt sensor, assuming that at mid stance it is only measuring the gravity acceleration. Results from a set of experiments involving several users walking at different inclinations on a treadmill confirm the feasibility of our approach. A statistical analysis of slope estimations shows in first instance that the technique is capable of distinguishing the different slopes of the walking surface for every subject. It reports a global RMS error (per-unit difference between actual and estimated inclination of the walking surface for each stride identified in the experiments) of 0.05 and this can be reduced to 0.03 with subject-specific calibration and post processing procedures by means of averaging techniques.

## 1. Introduction

Estimation of the slope of the walking surface during normal walking of people can be of interest in many different application fields. Just to name a few, it may be useful for localization purposes in ubiquitous computing environments for the deployment of location and activity based services. In this context, the inclination of the surface may help to accurately locate the subject by map-matching or from location transition tables as proposed in [1]. It is also of interest in the development of personal navigation systems, as it can help to estimate the geographical vertical position of the subject [2] or to correct the drift present in estimations as a consequence of the displacement over ramps [3]. Another field of interest is the clinical environment. Current health policies are concerned with the relation between physical activity during daily living and the physical [4] and psychological health of people. In this context, the inclination of the ground may be used to improve the assessment of the walking activity by considering whether subjects walked on level or inclined surfaces.

Kinematic estimation of gait performance using body worn sensors has been a main concern of research for the last decade [5]. Different magnitudes related to gait displacements and velocities have been accurately estimated from different sensor set-ups and signal processing methods.

**Figure 1.**
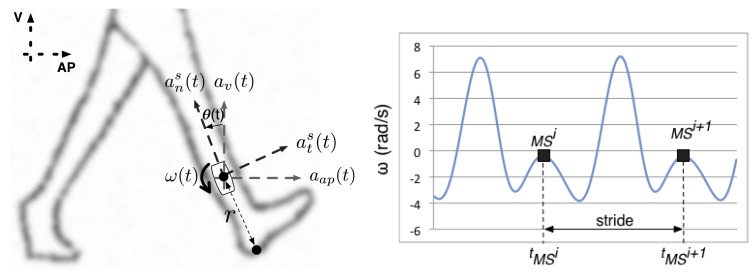
Sensor placement (**left**). Normal and tangential accelerations ( ${\text{a}}_{\text{n}}^{\text{s}}(\text{t}),{\text{a}}_{\text{t}}^{\text{s}}(\text{t})$) and rotation speed (*ω*(*t*)) are sampled from an inertial sensor attached to the external face of the shank, at an intermediate point between the knee and the ankle. Local maxima of lower lobes of shank rotation speed are used to segment the whole signal into single strides (right). MS stands for Mid Stance.

In particular, reference [6] reports a method valid for walking velocity estimation in a stride-by-stride sense. They apply strapdown integration techniques over the signals sampled from a shank-mounted inertial sensor (see Figure 1, left), composed of a biaxial accelerometer that measures the shank normal and tangential accelerations [ ${\text{a}}_{n}^{s}(t),{\text{a}}_{t}^{s}(t)$] and a gyroscope that measures the shank angular speed [*ω*(*t*)]. Strides are segmented at local maxima of lower lobes of shank angular speed (mid-stance events, Figure 1, right), where the shank is supposed to be parallel to the gravitational vector. For each stride, vertical and antero-posterior accelerations [*a_v_*(*t*), *a_ap_*(*t*)] in the global coordinate frame are estimated in first instance from the sampled signals. Vertical and antero-posterior displacements can then be estimated using integration procedures (initial vertical and antero-posterior velocities were supposed to be null), allowing one to compute the walking velocity, and other secondary parameters such as the stride length or the walked distance. This approach takes advantage of the inverted pendulum-like behaviour of the stance leg during walking to segment the gait cycle and to estimate the initial conditions for integration.

While this method has shown to accurately estimate the walking velocity, it did not work when used to estimate the slope of the walking surface. Reasons for this were discussed by the authors in the original work, addressing as the main issue that the shank, and therefore the sensor, may be not aligned with the gravity vector at mid-stance time when walking over sloped surfaces, and thus any deviation between them should be considered. Otherwise an offset would be introduced in the calculations leading integration procedures to unwanted results when used to estimate the slope of the walking surface.

In this paper we propose a novel approach for the estimation of the slope of the walking surface from a shank mounted inertial sensor. It mainly builds on the approach proposed in [6], solving the addressed issues by estimating the actual inclination of the sensor at mid-stance events by using the incorporated accelerometer as a tilt sensor.

The motivation for this approach is that the pendulum-like behaviour of the stance leg during normal walking [6] should allow to compute normal [***a****_n_*(***t***)] and tangential [***a****_t_*(***t***)] accelerations at any point of the shank from the general Equations (1) and (2), *r* being the distance from the point selected in the shank to the floor (length of the pendulum). At mid stance, ***a****_t_*(***t***) becomes zero, as shank angular rotation reaches a local maximum. On the other hand, ***a****_n_*(***t***) depends mainly on the shank rotation speed, expressed in quadratic form. If shank rotation speed is low at mid stance, as advanced in [6], normal acceleration should become negligible at that time. As a consequence, under these assumptions, sampled accelerations [ ${\text{a}}_{\text{n}}^{\text{s}}(\text{t}),{\text{a}}_{\text{t}}^{\text{s}}(\text{t})$] should only be due to the gravitational acceleration at mid stance:

(1) $$a_{t}(t)=\frac{d\omega (t)}{dt}$$

(2) $$a_{n}(t)=\omega {\left(t\right)}^{2}\times r$$

To validate the approach we have conducted a set of experiments involving several users walking on a treadmill set at different inclinations. Data from these experiments were used in first instance to validate the foundations of the method and to provide performance results in terms of the estimation error, precision and accuracy. This data was also used to analyze how subject-specific calibration procedures using data gathered from walking on surfaces of known inclination can be used to improve the accuracy of estimations. As a final study, we show what performance can be expected from the method when such calibration procedures are combined with averaging techniques aimed to reduce the variability of raw estimations.

Other state-of-the-art works have addressed the estimation of the slope of the walking surface from body mounted inertial sensors. For instance, an approach based on the tilt of a foot-attached accelerometer during zero velocity intervals is proposed in [2]. Reference [7] reports about a method based on strapdown integration techniques from a foot mounted inertial sensor. Reference [8] deals with the problem using a neural network as an estimator that processes acceleration signals from a sensor placed on the waist of the subject. Taken together with the aforementioned and perhaps other similar works, this paper contributes to extend the set of techniques useful to estimate the slope of the walking surface from body-mounted inertial sensors, showing the performance that can be expected from the approach.

## 2. Estimation of the Slope of the Walking Surface

The process mainly reproduces the original work by [6]. Gait signals are segmented by searching for local maxima of lower lobes of the shank angular rotation speed (Figure 1, right). A zero phase low pass filter with a cut-off frequency of 4 Hz is previously used to remove false local maxima in the sampled signal. Vertical and antero-posterior accelerations (*a_v_*(*t*), *a_ap_*(*t*)) in the global coordinate frame are estimated in first instance (Equations (3) and (4), g being the gravity acceleration) using the sampled normal and tangential accelerations ( ${\text{a}}_{n}^{s}(t),{\text{a}}_{t}^{s}(t)$) and the inclination of the sensor (*θ*(*t*), whose estimation is addressed later). These equations suppose in fact a transformation from the reference frame of the sensor to the global reference frame. Vertical and antero-posterior displacements (*S_v_, S_ap_*) can then be estimated (Equations (5)–(8)). Slope estimation (Θ) is then straightforward (Equation (9)):

(3) $$a_{v}(t)={\text{a}}_{n}^{s}(t)\cos\theta (t)+{\text{a}}_{t}^{s}(t)\sin(t)-g$$

(4) $$a_{ap}(t)=-{\text{a}}_{n}^{s}(t)\sin\theta (t)+{\text{a}}_{t}^{s}(t)\cos(t)$$

(5) $$V_{v}(t)=\int_{t_{MS^{i}}}^{t}a_{v}(\tau )d\tau$$

(6) $$S_{v}(t)=\int_{t_{MS^{i}}}^{t_{MS^{i+1}}}V_{v}(t)dt$$

(7) $$V_{ap}(t)=\int_{t_{MS^{i}}}^{t}a_{ap}(\tau )d\tau$$

(8) $$S_{ap}=\int_{t_{MS^{i}}}^{t_{MS^{i+1}}}V_{ap}(t)dt$$

(9) $$\Theta =\frac{S_{v}}{S_{ap}}$$

As proposed by [6], *θ*(*t*) can be estimated from the gyroscope signal (Equation (10)). However, the initial inclination of the sensor at the beginning of the stride (***θ_MS^i^_***, supposed null in [6]) must be considered. For such purpose, we use the accelerometer as a tilt sensor at mid stance events (Equation (11)) as proposed in [9]:

(10) $$\theta (t)=\int_{t_{MS^{i}}}^{t}\omega (\tau )d\tau +\theta_{MS^{i}}$$

(11) $$\theta_{MS^{i}}=\arctan\left(\frac{{\text{a}}_{t}^{s}(t_{MS^{i}})}{{\text{a}}_{n}^{s}(t_{MS^{i}})}\right)$$

## 3. Experiments and Data Analysis

Six adult subjects (four male, two female) without apparent impaired mobility were involved in the experiments after giving their informed consent. They were asked to walk about 100 m on a treadmill without holding its handlebars at seven different inclinations (0.14, 0.10, 0.06, 0.02, −0.02, −0.05, −0.09). These inclinations were measured as the change in the global reference frame between the vertical position of the rear and the front of the treadmill per unit of change in the horizontal position of both parts of the device.

For such purpose we have used a quasi-fixed treadmill. Only two inclinations were available by the device, using an incorporated lever (±0.02) (the device did not allow 0 inclination). The rest of inclinations were forced by introducing metallic planches at the front or at the rear.

The treadmill speed was fixed at 4 km/h (1.11 m/s) for all the experiments. An Xsens IMU was attached to the right shank of the subjects. Only accelerations in the sagittal plane (sampled at 100 Hz) and angular velocity around the medio-lateral shank axis were used for the calculations.

For each experiment, strides were identified from mid stance events. Then the slope of the walking surface was estimated for each stride. As a result of the experiments, we collected seven sets of estimations of the slope of the walking surface (one for each inclination of the treadmill) for each of the six subjects. Accelerations and rotation speed of the sensor at mid stance events were analyzed in first instance to experimentally assess the foundations of our work.

Raw estimations were analyzed in terms of Root Mean Estimation Error (RMSE), accuracy and precision to assess the performance of the proposed technique. Estimation errors were defined as the difference between the estimated and actual inclination of the walking surface (treadmill inclination). Accuracy was defined as the averaged estimation error. Precision was defined as the averaged standard deviation of estimations errors.

RMSE, accuracy and precision were sometimes analyzed independently for each user. In this case, we have used all the estimations available for that user at the seven different inclinations of the treadmill. Other times RMSE, accuracy and precision were analyzed in global terms. In this case, we processed all the estimations from all the users at all the inclinations of the treadmill.

The correlation coefficient of Pearson between the actual and estimated inclination of the slope of the walking surface was also calculated for each user considering all the estimations at the different inclinations of the walking surface for that user.

We have analyzed the effect over the accuracy of the technique of a linear correction of raw estimations using data gathered from experiments at two or more slopes. For such purpose we have supposed that raw estimations (*S_e_*) are related to actual inclination (*S_a_*) of the walking surface by a linear relation: *S_e_* = *S_a_* * *p* + *f, p* and *f* being the slope and bias of the linear model. So, using regression techniques to learn this relation from experiments at two or more different slopes, we have estimated the actual slope of the walking surface by correcting raw estimations using the inverse relation: *S_a_* = (*S_e_* − *f*)/*p*.

We have also considered that estimations are mainly affected by random white noise and thus we have analyzed the effect of averaging techniques over the dispersion of raw estimations. However, given that normal walking usually evolves over surfaces of changing slope, averaging should be performed over a reduced set of consecutive strides to adapt to such changes. As a consequence, we have implemented this averaging by means of a moving average filter, which is known to be an optimal filter for reducing random white noise in signals while keeping the sharpest step response [10].

Several statistical tests were used to analyze the data [11] at different stages. A one-way ANOVA analysis combined with the honestly significant difference of Tukey [12] was used to find significant differences in the mean of different groups of estimations. We have used this test to find whether mean estimations at different inclinations of the slope of the walking surface were significantly different among them for a given user. We have also used an ANOVA test to compare the accuracy of raw and calibrated estimations. A F-test for equal variances was used to find significant differences in the precision of the raw and averaged estimations.

## 4. Results and Discussion

### 4.1. Execution of Experiments

No relevant circumstances were observed during the execution of the experiments. The exception was Subject 3, who could not adapt to treadmill walking and showed a noticeably irregular pace, needing sometimes to tightly hold the handlebars of the treadmill during the experiments to avoid falling. As a consequence, although we have considered this subject for our analysis, his results are of an arguable significance about the performance of the technique. In this way, we can suggest that variations from patterns of normal gait may affect the performance of the method, which potentially may limit the applicability of the method to normal walking.

The number of strides varied for each subject and experiment (88 ± 8, 80 ± 4, 96 ± 5; 88 ± 7, 77 ± 8 and 100 ± 4, respectively, for subjects 1 to 6). The magnitude of the acceleration vector at mid stance from sampled normal and tangential accelerations [ ${\text{a}}_{n}^{s}(t),{\text{a}}_{t}^{s}(t)$] reported a value of 9.83 ± 1.5 m/s^2^, quite close to the expected value of 9.8 m/s^2^. We have also observed that five samples later/before from the identified maximum (only 5 hundredths of a second later/before in time) the magnitude of the acceleration vector is, respectively, 10 ± 1.4 m/s^2^ and 9.19 ± 1.4 m/s^2^. So, in our opinion, deviations from the expected value of the magnitude of the acceleration vector at mid stance are explained by inaccuracies in the timing of the physical event from the gyroscope signal, always imprecise to some extent.

### 4.2. Raw Estimations Analysis

**Figure 2.**
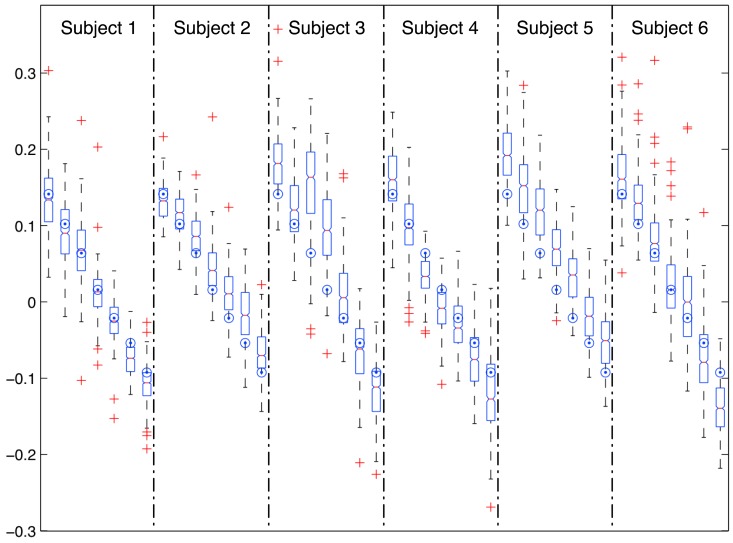
Raw estimations of the slope of the walking surface for the six subjects at the seven treadmill inclinations (0.14, 0.1, 0.06, 0.02, −0.02, −0.05, −0.09, from left to right for each subject). Big dots show the actual inclination of the treadmill for each experiment.

Figure 2 shows the distribution of raw estimations for each subject and treadmill inclination. Boxes show the dispersion and the central tendency (median) of estimations for each subject/treadmill inclination. Big dots show the actual treadmill inclination for each group of estimations.

Raw estimations for every subject and treadmill inclination are summarized in Table 1, including average and standard deviation of estimations and linear correlation coefficients (Pearson) and Root Mean Squared Error between estimations and actual treadmill inclination.

Root Mean Squared Errors of raw estimations range from 0.04 to 0.07, reporting a global RMSE between the actual and predicted treadmill slope of 0.05 for all the experiments. This means that in a general sense we should expect an average raw estimation error of about 0.05 for unconstrained subjects and walking surfaces. However, this average estimation error will differ for different users and slopes of the walking surface, as it statistically depends on both factors. In any case, our experimental results show that this variation is moderated and contained in the range from 0.04 to 0.07.

Accuracy (Table 2, first column) and precision (Table 2, fourth column) of the technique were analyzed from estimation errors. Global accuracy and precision are 0.013 and 0.052 respectively.

An ANOVA test, separately run for each individual, shows that mean raw estimations at every slope are significantly different from mean raw estimations at the remaining slopes.

We have found a strong linear relation for each subject between raw estimations and actual slopes of the walking surface (linear correlation coefficient of Pearson is about 0.9 for all the subjects as shown in Table 1). We have observed, on the other hand, that raw estimations show a high dispersion (low precision). These facts motivated us to analyze ways to improve raw estimations by means of linear calibration procedures and averaging techniques, with the results detailed next.

### 4.3. Improving Raw Estimations

In first instance, we have analyzed the effect of calibration procedures over the accuracy of the technique using a linear model that relates raw estimations to actual inclinations of the walking surface learned from experiments at two or more slopes. Table 2, column 2, shows the accuracy of corrected estimations at slopes of 0.14, 0.06, 0.02, −0.02, −0.09 when the experiments at slopes of 0.1 and −0.05 were used to define the linear model for the correction. Table 2, column 3 shows the accuracy of corrected estimations at slopes of 0.14, 0.06, −0.02, −0.09 when experiments at slopes of 0.1, 0.02 and −0.05 were used to define the linear relation. All cycles of the experiments selected for calibration were used to define the linear model. An ANOVA analysis shows that the accuracy is significantly improved in both cases, confirming that simple linear calibration procedures with experiments at a few different inclinations of the walking surface help to improve the accuracy of estimations.

Secondly, we have analyzed the effect of applying a moving average filter with a kernel of length five to raw estimations. Table 2, column 5 shows that filtered estimations (F-test for equal variances) present a significant improvement in precision if compared with the precision of raw estimations (column 4). The global improvement in precision is by a factor of 1.7, a consistent value with our expectations (moving average filters reduce random noise by a factor equal to the square root of the length of the kernel of the filter [10]).

**Figure 3.**
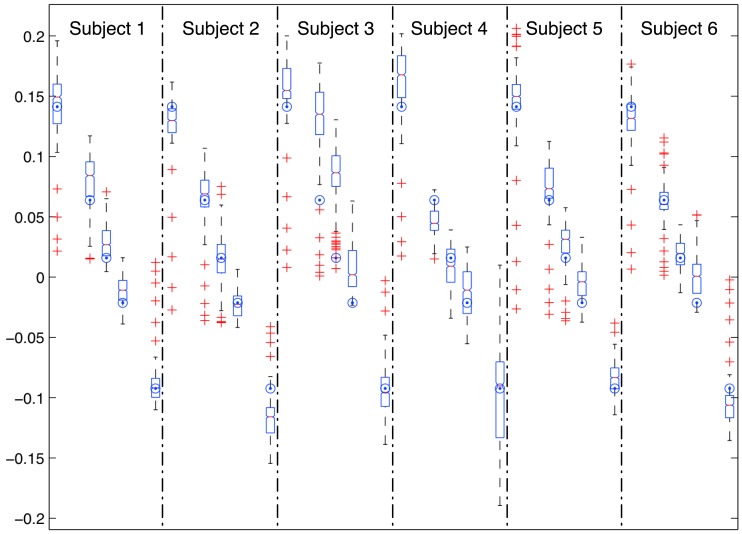
Corrected estimations for the six subjects at the inclinations (0.14, 0.06, 0.02, −0.02, −0.09, from left to right for each subject) using a linear model for each user defined from experiments at 0.1 and −0.05 and applying a moving average filter to raw estimations. Big dots show the actual inclination of the treadmill for each experiment.

As a final test of the expected performance of the technique with the proposed modifications, we have corrected raw estimations applying the mentioned moving average filter and a linear correction from experiments at 0.1 and −0.05. Figure 3 and Table 3 show the results. RMSE are in the range of 0.02 and 0.05 for all the users, 0.03 being the global RMSE. Global accuracy and precision are 0.008 and 0.032 respectively.

## 5. Conclusions/Outlook

In this paper we propose a novel approach for the estimation of the slope of the walking surface during normal walking by means of a shank mounted inertial sensor. For such purpose we investigate the feasibility of solving the issues posed by a similar approach [6]. As explained in their work, the main drawback of their approach is that the inclination of the shank (and thus the inclination of the sensor) may be different from null at mid-stance events. This inclination must be considered in the set of initial conditions used to calculate the actual inclination of the sensor at any time during the stride, in order to project the normal and tangential sampled accelerations over the vertical and antero-posterior ones. Otherwise, an offset is introduced in the calculations leading to incorrect results in the estimation of the slope of the walking surface [13].

Our proposal, inspired by the biomechanical characteristics of human gait, is to estimate the inclination of the sensor at mid-stance events by using the accelerometer as a tilt sensor. This approach has shown encouraging results in [9] when used to estimate the geographical vertical position of a subject during unconstrained walking from a similar sensor setup. To assess the performance of the approach we have designed a set of experiments involving different users walking on a treadmill at a representative range of inclinations.

From these experiments, we have given in first instance empirical support for our assumption about the negligible effect of normal and tangential shank accelerations over the accelerations registered at the sensor at mid stance events,

Taking the experimental raw estimations for each subject in isolation, we have found that mean slope estimation for every treadmill inclination is significantly different from mean estimations for the remaining treadmill slopes. This allows concluding initially that for a single user the technique is capable of differentiating in average terms walking surface inclinations at the precision of our experimental setup (about 0.04).

Raw estimations report a global RMSE of 0.05. However, we have shown that, on the one hand, the accuracy can be significantly improved if necessary by means of subject-specific linear calibration procedures from data gathered from two or more experiments at different slopes of known inclination (two experiments at only one slope of known inclination would be enough to define the linear model if we walk in both directions: one experiment ascending the slope and the other descending the slope). Although this can be seen as a limitation of the approach, we would like to stress that calibration is only necessary when we need an improved accuracy beyond that which can be obtained using the basic method. On the other hand, as expected from a method like the proposed in this paper eventually influenced to some extent by random errors, averaging techniques can be used to reduce the dispersion of raw estimations. Given that in the context of normal walking averaging should be applied locally to handle potential changes in the slope of the walking surface, the price to pay for this is that from a practical point of view several consecutive strides at the same inclination would be necessary to get more precise estimations, but we show how a few strides can be enough for a significant improvement.

As a final sample we have applied such techniques to the experimental data allowing to improve the performance of the technique (global RMSE is reduced from 0.05 to 0.03).

As a final remark, we found it very difficult to compare the performance of our method with the performance of other similar methods [2,7,8] from the results reported by the authors in the published papers. Anyway, we think that our method could be considered preferable from the usability point of view in some circumstances. In first instance, the shank can be a friendlier segment to attach a sensor to than the foot [2,7] as in many circumstances it could be very unsightly to wear a sensor onto the shoe or it could be uncomfortable to hide it under the shoe. However, to attach a sensor to the shank using elastic bands could be easier and trousers or socks could easily hide the sensor from the external view. On the other hand, our approach is based on biomechanical principles rather than on black box approaches (neural networks) and does not require exhaustive initial training procedures to tune the estimation method to the user [7].

## Figures and Tables

**Table 1.** Numerical results (raw estimations) of the experiments. S_n_ stands for Subject n. Average and standard deviation of estimations are shown for every subject and treadmill inclination. Linear correlation coefficient values (correlation coefficient of Pearson) between estimations and actual inclination of the treadmill and root mean squared error (RMSE) of estimations are also shown for each subject.

	**Treadmill Inclinations**							**Corr. Coef.**	**RMSE**
	**0.14**	**0.1**	**0.06**	**0.02**	**-0.02**	**-0.05**	**-0.09**		
S_1_	0.13 ± 0.04	0.09 ± 0.04	0.07 ± 0.05	0.01 ± 0.04	−0.03 ± 0.03	−0.07 ± 0.02	−0.11 ± 0.03	0.92	0.04
S_2_	0.13 ± 0.03	0.12 ± 0.03	0.09 ± 0.03	0.04 ± 0.04	0.01 ± 0.03	−0.01 ± 0.04	−0.07 ± 0.03	0.89	0.04
S_3_	0.18 ± 0.04	0.12 ± 0.04	0.15 ± 0.06	0.1 ± 0.06	0.01 ± 0.05	−0.07 ± 0.04	−0.12 ± 0.04	0.86	0.07
S_4_	0.16 ± 0.04	0.1 ± 0.05	0.03 ± 0.03	−0.01 ± 0.03	−0.03 ± 0.04	−0.07 ± 0.04	−0.12 ± 0.06	0.9	0.05
S_5_	0.2 ± 0.04	0.15 ± 0.05	0.12 ± 0.04	0.07 ± 0.04	0.03 ± 0.04	−0.02 ± 0.04	−0.05 ± 0.04	0.91	0.06
S_6_	0.17 ± 0.05	0.13 ± 0.04	0.08 ± 0.05	0.02 ± 0.05	0 ± 0.06	−0.07 ± 0.05	−0.14 ± 0.04	0.89	0.05

**Table 2.** Accuracy and precision of raw and corrected estimations. Columns 1 and 4: accuracy and precision of raw estimations. Columns 2 and 3: accuracy of estimations for a calibration using experiments at two (0.1, −0.05) and three (0.1, 0.02, −0.05) slopes respectively. Columns 5: Precision of estimations after applying a moving average filter to raw estimations. S_n_ stands for Subject n.

	**Accuracy**			**Precision**	

	**Raw**	**Calibrated (2 exp.)**	**Calibrated (3 exp.)**	**Raw**	**Filtered**

S_1_	−0.01	0.01	0	0.04	0.02
S_2_	0.02	−0.01	−0.01	0.03	0.02
S_3_	0.03	0.03	0	0.05	0.03
S_4_	−0.01	0	0	0.04	0.03
S_5_	0.05	0.01	0	0.04	0.02
S_6_	0.01	0	0	0.05	0.02

**Table 3.** Corrected estimations using a linear model for each user defined from experiments at 0.1 and −0.05 and applying a moving average filter to raw estimations. Average and standard deviation at every inclination but those used for calibration purposes are shown. Root Mean Squared Error (RMSE) of estimations is also shown for each subject. S_n_ stands for Subject n.

	**Treadmill Inclinations**					**RMSE**

	**0.14**	**0.06**	**0.02**	**−0.02**	**−0.09**	

S_1_	0.14 ± 0.03	0.08 ± 0.02	0.03 ± 0.01	−0.01 ± 0.01	−0.09 ± 0.02	0.02
S_2_	0.12 ± 0.03	0.07 ± 0.03	0.01 ± 0.02	−0.02 ± 0.01	−0.12 ± 0.02	0.03
S_3_	0.16 ± 0.03	0.13 ± 0.04	0.08 ± 0.03	0.01 ± 0.02	−0.09 ± 0.02	0.05
S_4_	0.16 ± 0.03	0.05 ± 0.01	0.01 ± 0.02	−0.01 ± 0.02	−0.1 ± 0.04	0.03
S_5_	0.15 ± 0.04	0.07 ± 0.02	0.03 ± 0.02	−0.01 ± 0.02	−0.08 ± 0.01	0.03
S_6_	0.13 ± 0.03	0.06 ± 0.02	0.02 ± 0.01	0 ± 0.02	−0.1 ± 0.0	0.02
